# Can Machine Learning and PS-InSAR Reliably Stand in for Road Profilometric Surveys?

**DOI:** 10.3390/s21103377

**Published:** 2021-05-12

**Authors:** Nicholas Fiorentini, Mehdi Maboudi, Pietro Leandri, Massimo Losa

**Affiliations:** 1 Department of Civil and Industrial Engineering, Engineering School of the University of Pisa, Largo Lucio Lazzarino 1, 56126 Pisa, Italy; pietro.leandri@ing.unipi.it (P.L.); losa@ing.unipi.it (M.L.); 2 Institute of Geodesy and Photogrammetry, Technische Universität Braunschweig, Bienroder Weg 81, 38106 Braunschweig, Germany; m.maboudi@tu-bs.de

**Keywords:** International Roughness Index (IRI), Machine Learning Algorithms (MLAs), Persistent Scatterer Interferometric Synthetic Aperture Radar (PS-InSAR), radar interferometry, laser profilometric surveys, Pavement Management Systems, road roughness

## Abstract

This paper proposes a methodology for correlating products derived by Synthetic Aperture Radar (SAR) measurements and laser profilometric road roughness surveys. The procedure stems from two previous studies, in which several Machine Learning Algorithms (MLAs) have been calibrated for predicting the average vertical displacement (in terms of mm/year) of road pavements as a result of exogenous phenomena occurrence, such as subsidence. Such algorithms are based on surveys performed with Persistent Scatterer Interferometric SAR (PS-InSAR) over an area of 964 km^2^ in the Tuscany Region, Central Italy. Starting from this basis, in this paper, we propose to integrate the information provided by these MLAs with 10 km of in situ profilometric measurements of the pavement surface roughness and relative calculation of the International Roughness Index (IRI). Accordingly, the aim is to appreciate whether and to what extent there is an association between displacements estimated by MLAs and IRI values. If a dependence exists, we may argue that road regularity is driven by exogenous phenomena and MLAs allow for the replacement of in situ surveys, saving considerable time and money. In this research framework, results reveal that there are several road sections that manifest a clear association among these two methods, while others denote that the relationship is weaker, and in situ activities cannot be bypassed to evaluate the real pavement conditions. We could wrap up that, in these stretches, the road regularity is driven by endogenous factors which MLAs did not integrate during their training. Once additional MLAs conditioned by endogenous factors have been developed (such as traffic flow, the structure of the pavement layers, and material characteristics), practitioners should be able to estimate the quality of pavement over extensive and complex road networks quickly, automatically, and with relatively low costs.

## 1. Introduction

Road infrastructures offer a critical contribution to economic development and constitute the social fabric of a developed country [1]. Inadequately managed, maintained, and controlled roads constrain mobility, markedly increasing transportation operating costs, accident rates, and related human and property costs, and aggravate segregation, poverty, and poor health [2]. In many European countries, such as Italy, road authorities are supported by increasingly limited funds for road monitoring and inspection. Accordingly, in order to save as much money as possible, it is critical to quickly detect preventive road maintenance interventions. Considering that road authorities have to manage vast and heterogeneous networks, the development of network-scale tools is strongly demanded. Such tools should allow carrying out screening activities to identify potential infrastructural hazards and critical road sections. Therefore, screening tools allow road authorities to perform further in situ inspections over a restricted number of targeted sites, thus, saving time, money, and having a minimal impact on system serviceability. Non-Destructive High-Performance Techniques (NDT) are the basis of these activities and supply the Pavement Management Systems (PMS) with valuable data since they are characterized by high accuracy, reliability, speed of execution, and high coverage, and they can reduce the number of in situ inspections [3]. Generally, these techniques are suitable for assessing a specific aspect of the infrastructure, and multiple NDT-based surveys are required to obtain a comprehensive assessment of the asset conditions. As a result, more studies attempt to merge NDT-based outcomes in order to develop predictive models that should significantly reduce the number of surveys.

In the present paper, we focus on integrating two NDT-based techniques: the multi-temporal Synthetic Aperture Radar (SAR) interferometry, namely the Persistent Scatter Interferometric SAR (PS-InSAR), and the profilometric measurement of road roughness by the Laser Profiler. In the context of infrastructure monitoring and inspection, these surveys provide two pieces of information that may be correlated. Specifically, PS-InSAR allows for identifying surface motions and displacements on and around infrastructures, whereas profilometric measurements allow for assessing the quality of road pavements in terms of irregularities and roughness. Consequently, it is conceivable that subsidence and swelling of the road surface (detected by the PS-InSAR) may cause a significant damage and deficiencies of the pavement, affecting regularity (detected by the Laser Profiler). Nonetheless, these measurements are different. Indeed, road roughness is expressed generally in terms of [mm/m], whereas the PS-InSAR-based technique provides displacement ratios in terms of [mm/year]. The identification of a correlation between PS-InSAR and International Roughness Index (IRI) provides road authorities two pieces of information with a single NDT-based survey. Once the interferometric process has been carried out, the IRI of an extensive road network should be easily derived. To the best of our knowledge, few research investigated the validation of SAR-based products by profilometric measurements. We found one valuable and recent study by Meyer et al. [4], in which the authors developed a model that allows for predicting IRI employing amplitude (backscattered brightness) data of X-band High-Resolution COSMO Sky-Med SAR images. Similar research has been carried out by Suanpaga et al. [5]. The authors calibrated a logit model to predict the IRI by exploiting Phase Array type L-band Synthetic Aperture Radar (PALSAR) images. The authors found a significant correlation between the IRI measurement and SAR amplitude information. Finally, it is worth mentioning the recent study of Karimzadeh and Matsuoka [6], in which the authors proposed a methodology for reducing traffic jam effects on SAR backscattered amplitude data. They support that SAR-based products can be used efficiently for assessing the quality of road pavements and that results are comparable with an in situ road roughness measurement.

Our research aims to compare IRI measurements with the outcomes derived by the PS-InSAR technique applied to Medium-Resolution C-band Sentinel-1 SAR images in the context of Italian, rural, two-lane roads. We aim to check if PS-InSAR is sufficiently able to detect surface irregularities highlighted by a profilometric survey. The proposed methodology stems from two previous studies [7,8], in which we calibrated several Machine Learning Algorithms (MLAs) for predicting vertical deformation of road pavement surfaces, in terms of [mm/year], by linking PS-InSAR-based outcomes and several environmental parameters. This modeling phase was due to insufficient PS coverage on the road surface of two-lane rural roads. As a consequence of this modeling through MLAs, it was possible to recognize a clear association between PS-InSAR-based surface motions, environmental parameters, and, mostly, the surface motion at every point of the road pavement.

Therefore, starting from environmental data and medium-resolution SAR measurements of a large area, MLAs were calibrated for evaluating surface motions, and, then, in the present research, such outcomes have been integrated with profilometric surveys. The aim is to verify if road authorities can evaluate the quality of road pavements in terms of IRI by exploiting information related to SAR-based products.

## 2. State-of-the-Art

### 2.1. Monitoring Road Infrastructure by PS-InSAR-Based Surveys

The PS-InSAR technique is a multi-temporal SAR interferometry [9], in which practitioners overcome the drawbacks of temporal and geometrical de-correlation by exploiting long stacks of co-registered SAR images. A PS-InSAR outcome assumes the form of a point-based map that allows identifying surface displacement trends over time [10] along the Line-Of-Sight of the SAR sensor. Such points are the Persistent (or Permanent) Scatterers (PS), i.e., on-ground stationary items for which backscattered data (phase and amplitude) do not vary markedly among SAR images gathered over time. Starting from the master image, each new SAR acquisition expands PS displacement trends of a new value. At the end of the PS-InSAR process, the displacement values are averaged for each PS, thus, getting the average deformation velocity (or surface motion rate), usually displayed in terms of [mm/year]. Generally, the PS is identified by human-made structures and outcropping rocks. In addition, the Distributed Scatterers (DS) InSAR algorithm has been recently developed for improving the PS-InSAR-based approach [11,12,13], while PS are identified by single pixels with high back-scattered signals (human-made structures and outcropping rocks). DS are on-ground items that rely on medium or low backscattered signals and can only be identified if they constitute homogeneous groups of pixels large enough to allow statistical analysis. Accordingly, DS-InSAR can enlarge the field of applications of PS-InSAR, since DS can be extracted from large areas of bare soil, sparsely vegetated land, and debris/earth accumulations. Each DS is represented by a single point of measure. Nonetheless, it is characterized by the “effective area”, i.e., the extension of the area which each DS refers [14,15].

The highest performance of multi-temporal SAR interferometry is achieved in surveys over urbanized areas and infrastructures, where several PS should be detected [16,17].

The technique can be efficiently employed for detecting surface motion patterns in the context of slow or relatively slow movements due to human-related [18,19] or environmental-related activities, e.g., subsidence [20,21,22], sinkholes [23,24], and landslides [25,26,27,28,29,30]. Readers may find additional valuable sources in Reference [8].

The high capabilities of SAR sensors in terms of measurement reliability, accuracy, speed of execution, high coverage, and the possibility of data processing in near real-time and back in time have meant a bursting development of SAR-based products as high-performance NDT in monitoring and inspection activities of linear infrastructures. In the literature, there are several SAR-based applications, mainly PS-InSAR surveys, of infrastructure monitoring activities, e.g., road infrastructures [6,7,8,31,32,33,34,35], rail infrastructures [36,37,38,39,40], airport runways [41], and bridges [42,43,44,45]. In these research studies, the common objective is to identify critical infrastructural sections by processing SAR images and comparing these surveys with other techniques (e.g., leveling or GPS surveys). Furthermore, research has focused on planning activities of infrastructures, exploiting radar interferometry to recognize areas in which it is more appropriate to build infrastructures [35], and research on prevention activities, where the PS-InSAR survey is inserted in a Pavement Management System perspective [32].

The study of Ozden et al. [46] shows that radar interferometry improves the benefit/cost ratio of an infrastructural monitoring plan.

### 2.2. Measuring Road Roughness by Profilometric Sensors and the IRI

Ordinarily, the IRI is the parameter employed for evaluating the longitudinal irregularities, or road roughness, of a road pavement surface. The IRI is an aggregate metric evaluated by measuring the longitudinal profile of road pavement, according to the procedure defined by the World Bank in 1986 [47]. A valuable tutorial on how to compute the IRI can be found in the research of Sayers [48] and Loizos [49]. The IRI parameter estimation can be realized after performing profilometric measurements carried out on road pavements using specific laser devices. In the literature, there are dozens of studies completed on the use of laser profilometric measurements and related IRI calculations. Airport runways [50,51] as well as flexible [52,53,54,55], rigid [53], and composite pavements [52,53] have been analyzed. In general, in these studies, structural data of road pavements or surface distresses are correlated with IRI, with a common purpose of developing helpful regression models for infrastructure management and maintenance.

### 2.3. Machine and Deep Learning Algorithms in Road Pavement Management

It has been mentioned that a profilometric survey is an expensive activity in terms of money and time. With the development of robust computational machines and the advent of Machine and Deep Learning modeling, many researchers have focused their attention on developing advanced statistical models of pavement irregularities. The common purpose is to show that using such techniques allows for reliable results with a significant time saving. In the large majority of cases, road roughness models have been proposed for predicting the IRI parameter.

Table 1 systematically reviews some valuable works focused on this topic. It reports the Reference (Ref.), the Machine Learning or Deep Learning Algorithms implemented (Algorithms), the output dependent variable (Output), the road (Road), the type of pavement investigated (Pavement), and the data employed in the modeling (Data).

Some relevant aspects can be extracted from Table 1:

- IRI is the reference parameter for judging road pavement surface quality. This fact is likely due since IRI is recognized internationally, is widely used in Pavement Management Systems, is an objective measure (because it derives from standardized profilometric surveys), allows comparisons between infrastructural sections, and is a concise but explanatory metric;
- Traditional Artificial Neural Networks (i.e., the Multilayer Perceptron Neural Networks) are applied in most cases by obtaining outstanding performance. This technique is one of the first developed in the field of Machine Learning modeling and, considering that satisfactory results are generally obtained, the strategy has resisted over time. It continues to be significantly implemented and refined, e.g., by a Recurrent Neural Network and Radial Basis Function Neural Networks. There are sporadic studies in which the authors have achieved valuable results with other techniques, such as Random Forest, Boosted Regression Tree, and Support Vector Machine. Reasonably, academic researchers desired to move away from the steady application of Artificial Neural Networks by evaluating the performance of different algorithms that, in the field of pavement [70,71,72,73], transport [74,75], and road safety engineering [76,77,78,79], have led to reliable results;
- The Long-Term Pavement Performance open database provided by the Federal Highway Administration is extensively employed. This fact is a direct consequence of the characteristics of this database: open-source, already structured, heterogeneous, extensive, and allows comparing the outcomes among dozens of studies;
- Both rigid pavement structures (Join Plain Concrete pavements) and flexible pavement structures (asphalt or bituminous concrete layers overlying a base of granular material on a prepared subgrade) are examined, with a majority part focused on the study of the latter type. This is plausibly associated with the fact that flexible pavements are the most adopted worldwide;
- In almost all studies, climate and traffic data are gathered and implemented to predict the IRI. Structural and subgrade pavement data are often supplemented. Occasionally, information on the initial IRI value, age of the pavement, and surface distressed data are also added. Certainly, empirical knowledge plays an influential role. It is known that environmental factors, such as rainfall, days of frost, and days of extreme heat, and traffic flow factors could reflect in the regularity of a road pavement. Including information on the history of the IRI value, if available, may be beneficial, as well as information on the subgrade and superficial distresses. In any case, the more variables are inserted, the more complex and less interpretable the model will be. Machine and Deep Learning algorithms tend to overfit the data when trained with numerous variables. Consequently, a certain balance between sample size, number of input variables, and complexity of the resulting model should always be considered.

The first three aspects agree entirely with the findings of Marcelino et al. [63] published in November 2019. By reviewing six papers concerning MLAs in the field of road roughness prediction, the authors highlighted the same facts we found on the fourteen studies reported in Table 1. Therefore, the academic trend from November 2019 is not changed substantially.

## 3. Test Sites and SAR-Based MLAs

### 3.1. Study Area

**Figure 1:**
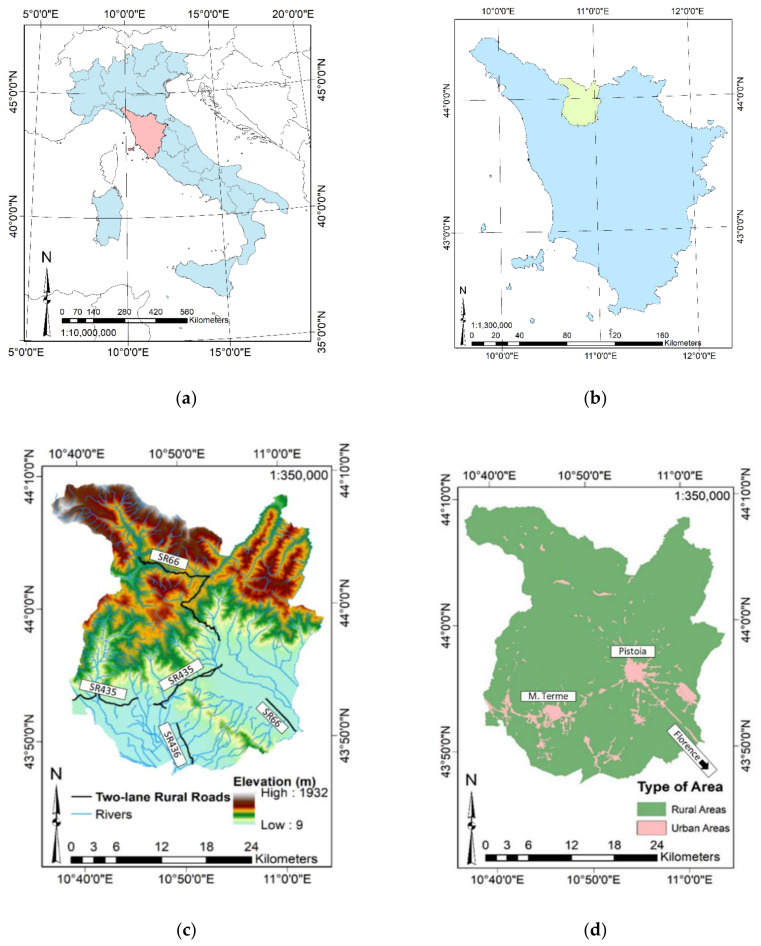
Study area: (**a**) Italy (light blue) and the Tuscany Region (pink). (**b**) Tuscany Region (light blue) and the Province of Pistoia (light green). (**c**) Province of Pistoia: elevation, river network, and two-lane rural roads crossing the area. (**d**) Province of Pistoia: rural and urban areas. The present figure has been adapted from Fiorentini et al. [8].

The present research is focused on three test sites located in the Province of Pistoia (Figure 1c,d), the Tuscany Region (Figure 1b), and central Italy (Figure 1a). For a comprehensive overview of the area, Figure 1c shows the topography of the area by a digital elevation map, the river network, and the stretches of two-lane roads managed by the Tuscany Region Road Administration (TRRA). These stretches are included in three regional roads, called SR435, SR436, and SR66. In the present research, two rural test sites have been selected from SR435 and one urban test site from SR66. Finally, Figure 1d reports the rural and urban areas. Mainly, it is possible to note two urban areas: the left one, located in the southern part of the Province, represents the city of Montecatini Terme, whereas the right one, in the central-eastern zone, represents the city of Pistoia. SR435 connects these two cities, whereas SR66 connects the city of Pistoia with the city of Florence, in the southeastern part of the study area.

**Figure 2:**
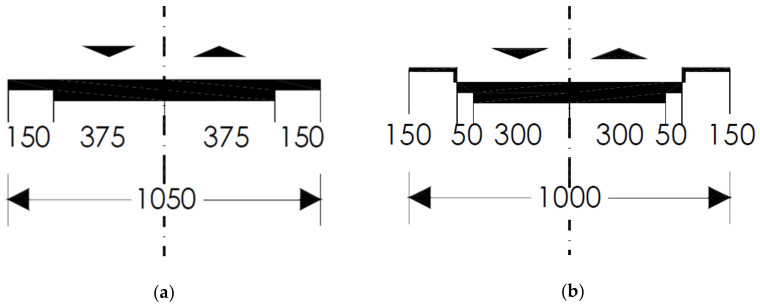
Italian two-lane road section (**a**) rural, road typology: “C1”; (**b**) urban, road typology: “E1”.

Such roads are monitored and managed by the TRRA, which provided all the required environmental data for developing the previously mentioned SAR-based MLAs [7,8]. Two-lane roads are defined in the Highway Safety Manual [80] as single carriageway roads with one lane for each travel direction. In Italy, such roads can cross rural or urban areas. Rural two-lane roads should have at least a lane width of 3.75 m, a paved shoulder of 1.50 m, a minimum planimetric radius with a curve of 118 m, and a maximum longitudinal slope of 7%. Urban two-lane roads should have at least a lane width of 3 m, a paved shoulder of 0.50 m, a sidewalk of 1.50 m, a minimum planimetric radius with a curve of 50 m, and a maximum longitudinal slope of 8%. Figure 2 reports the road section of both rural (Figure 2a) and urban (Figure 2b) road, according to Italian standards [81].

The present research is focused on both typologies of roads: test site 1 and test site 2 involve rural two-lane roads (typology “C1” for Italian standards), while test site 3 concerns an urban two-lane road (typology “E1” for Italian standards).

**Figure 3:**
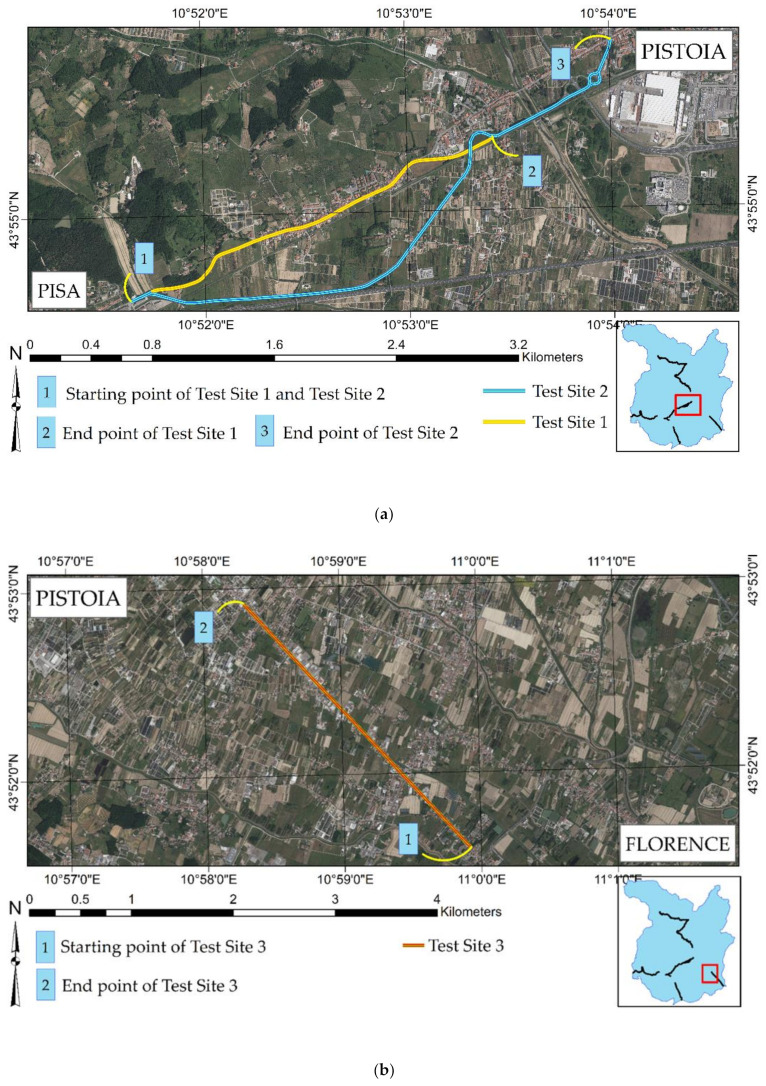
Test sites: (**a**) test site 1 and test site 2. (**b**) Test site 3.

Figure 3 shows the selected test sites. Each of them is represented by a stretch of two-lane road with a length that spans from 2.7 to 4.0 km. Test Site 1 (yellow line in Figure 3a) has a length of 2.7 km and is represented by the northern stretch of Figure 3a, from point 1 to point 2. This stretch is quite linear: starting from point 1, it follows the northern road up to point 2, where a road intersection is placed. Test site 2 (blue line in Figure 3a) has a length of 4 km and starts from the same point 1. The stretch moves along the southern road straight up to a 45° planimetric curve and then continues with a second linear stretch up to a very narrow, right curve. After point 2, it continues straight up to a roundabout intersection and ends shortly after (point 3). Point 3 is placed at the western boundary of the city of Pistoia. Furthermore, Figure 3a highlights where test site 1 and test site 2 are located concerning the Province of Pistoia (red rectangle above the light-blue map) and the travel directions (Pistoia and Pisa) that define the two profilometric routes.

Figure 3b represents test site 3 (red line). It is a road stretch of 3.3 km in length located in the southern part of Pistoia. It starts from a road intersection (point 1) and moves entirely in a straight line up to Pistoia’s southern boundary (point 2). Moreover, Figure 3b shows where test site 3 is located concerning the Province of Pistoia (red rectangle above the light-blue map) and the travel directions (Pistoia and Florence) that define the two profilometric routes.

**Figure 4:**
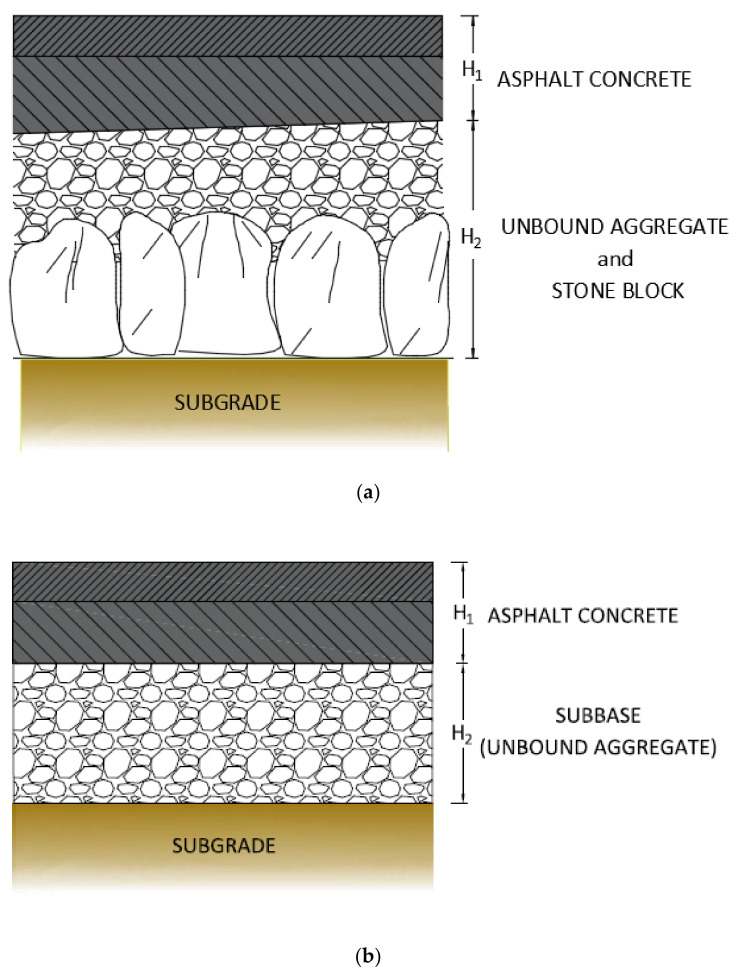
Pavement multilayer scheme (**a**) rural (test site 1 and test site 2). (**b**) Urban (test site 3).

In order to evaluate the scheme of the pavement multilayer (layer types and thicknesses) and the layer modulus ($E_{m}$), the test sites have been surveyed by a Falling Weight Deflectometer (FWD) and Ground Penetrating Radar (GPR). Moreover, two traffic monitoring stations have been employed to evaluate the Average Annual Daily Traffic (AADT) and the percentage of trucks (heavy vehicles). Figure 4 reports the pavement multilayer scheme of both rural (Figure 4a) and urban (Figure 4b) pavements. They reflect the pavement multilayer scheme of test site 1, test site 2 (rural), and test site 3 (urban).

Figure 4 highlights that the all the investigated pavements are flexible, composed by asphalt concrete layers (surface course, binder course, base course), unbound granular sub-base course, and compacted subgrade. Moreover, in the rural two-lane roads, a layer of stone block has been recognized below the sub-base layer. Table 2 quantifies the thickness of layers ($H_{1}$ and $H_{2}$ in Figure 4), and reports the actual range of lane width, pavement shoulder width, sidewalk width, AADT, percentage of trucks, $E_{m}$ (for asphalt concrete layers, subbase course, and subgrade course), and the subgrade classification for both rural and urban two-lane roads (according to the American Association of State Highway and Transportation Officials, AASHTO, Soil Classification System, Washington, DC, USA).

Table 2 shows that the current geometric characteristics of the test sites confirm that the road sections comply with Italian standards. The average width of the paved area is about 10 m (in urban roads, the sidewalks are also included). The traffic flow is significantly greater in the urban area than in the rural one (78.6% higher), but the dominant percentage of heavy vehicles cross the rural area (at least double than the urban area), confirming that heavy vehicles prefer avoiding the city center. Both in rural and urban pavements, we found poor geotechnical conditions of the subgrade for its use as a road subgrade (highly compressible silty soils and clayey soils).

It is worth mentioning that all the previously mentioned information (excluding the local geotechnical condition) is considered to be endogenous factors of the analyzed infrastructures. Accordingly, even if they could have a significant impact on road pavement surface regularity, we did not consider them as input features of MLAs. As previously stated, the present research aim is to evaluate how road regularity is driven by exogenous events of infrastructures, i.e., by natural events such as subsidence and uplifts, which are not linked with endogenous factors. Certainly, endogenous factors are relevant. They are currently under analysis as input of other MLAs. Once these MLAs conditioned by endogenous factors have been calibrated, practitioners should be able to estimate the quality of pavements comprehensively, concerning both exogenous and endogenous factors. Accordingly, the resilience of the infrastructure should be quantified appropriately.

### 3.2. SAR Data and InSAR Processing

In the present research, we referred to the PS-InSAR process steadily performed by the TRE Altamira (https://site.tre-altamira.com, accessed on 12 February 2020), Milan, Italy, on behalf of the Tuscany Region, every six days (corresponding to the temporal resolution of Sentinel-1). The PS-InSAR process is provided for the entire Tuscany Region. Moreover, such data is provided free of charge, in a Shapefile format, by the “Geoportale Lamma” (https://geoportale.lamma.rete.toscana.it/difesa_suolo/#/viewer/openlayers/326, accessed on 25 July 2020). It is worth noting that “Geoportale Lamma” is the first worldwide example of a regional-scale monitoring system based on continuous PS-InSAR processing [82]. In the present PS-InSAR processing, a stack of 210 co-registered SAR images (in ascending orbit) was employed. This stack covers a period from 12 December 2014 to 24 August 2019. The subsidence impact computed reached an intensity of 29.6 mm/year, mostly in the city center of Pistoia, while uplifts assume maximum values equal to 11.1 mm/year over the surrounding areas.

These surface motion estimations have been validated in Reference [22] by Global Navigation Satellite System (GNSS) measures, considered as external ground truth information. It has been shown that the variation in the vertical displacements detected by the Sentinel-1 data before and after the GNSS correction is about 1.0 mm/year. Accordingly, we considered the computed PS-InSAR velocities trustworthy to be used for calibrating MLAs.

**Figure 5:**
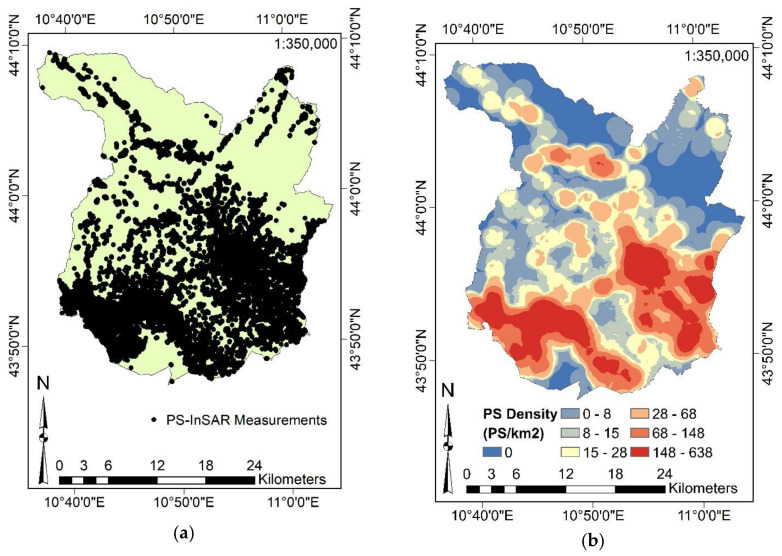
Persistent Scatterer (PS) measurements over the study area (52,257 PS): (**a**) Localization of PS. (**b**) PS density. Adapted from Fiorentini et al. [8].

After the PS-InSAR process, in order to guarantee the validity of estimations, we selected 52,257 PS with a coherence greater than 0.9 over the entire study area. The localization of PS over the study area is represented in Figure 5a. Such a figure does not describe the PS velocities given the high number of overlapping PS in urban areas. Figure 5b shows the PS density. As expected, considering Figure 1d, most of the PS are located in the urban areas of the province of Pistoia, as well as in the main road connections. By using a scale of representation of 1:350,000, it is not possible to clearly appreciate any surface movements related to subsidence or uplifts. Therefore, the aim of the present figure is to show readers where all the PS employed are localized as output targets in the MLAs.

**Figure 6:**
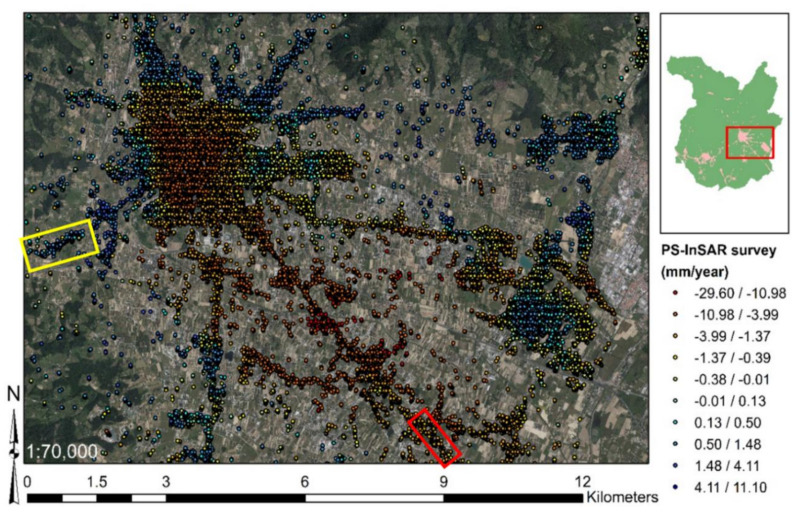
Map of surface motion over the city of Pistoia, based on “natural breaks” (ESRI ArcGIS 10.5). Adapted from Fiorentini et al. [8].

In order to evaluate the intensity of surface motions, readers can refer to Figure 6. This figure reports the previously mentioned subsidence and uplift effects affecting the city of Pistoia. The observed surface motion velocities are shown in a scale with green for the stability range, hot colors for negative velocities, and cold colors for positive velocities. The classification has been made by ESRI ArcGIS 10.5 [83], according to the “natural breaks”. Additionally, test sites are highlighted (test site 1 and test site 2 in the yellow rectangle, and test site 3 in the red rectangle). It is possible to verify that, in the test sites area, there are numerous PS. This aspect supports the use of PS-InSAR measurements to map subsidence and uplift the infrastructure due to natural causes.

### 3.3. SAR-Based MLAs and Estimated Vertical Displacements along with Test Sites

In our previous works [7,8], we calibrated some MLAs for predicting and mapping the average vertical displacement of the road pavement surface as a result of exogenous phenomena occurrence. Exogenous events are defined as external factors to the infrastructure. However, they may affect infrastructure conditions and their serviceability and are represented by extreme natural events, such as landslides, subsidence, and floods. It is known that such phenomena can be linked to environmental parameters of a territory, such as topological, geomorphological, geomorphometric, and hydrological features [84,85,86,87]. Therefore, by using MLAs, we aimed to correlate PS-InSAR-based surface motion estimates and several environmental parameters. Specifically, in Reference [7], a Classification and Regression Tree algorithm (CART) [88] has been calibrated, whereas, in the next study [8], we extended the procedure by adding the calibration of a Random Forest (RF) algorithm [89], a Support Vector Machine (SVM) for Regression [90,91], and a Boosted Regression Tree (BRT) algorithm [92,93].

The average velocity of each PS (in terms of mm/year) has been considered the target output of the MLAs. It is important to underline that, during the calibration of Machine Learning Algorithms, it was not essential that PS were over the road. In order to map the subsidence in each point of the surface (the whole Province of Pistoia), we performed an environmental analysis. The input features of such MLAs are 29 environmental-based factors related to the study area: elevation, aspect, slope, curvature, convergence index, slope-length, topographic position index, vector ruggedness index, terrain ruggedness index, average yearly rainfall, topographic wetness index, stream power index, river density, distance from rivers, earthquake susceptibility, distance from landslides, diffusive and direct yearly solar radiation, wind exposition, percentage of sand, silt, clay, and organic content in the subsoil, drainage capacity of the soil, flood susceptibility, erosion susceptibility, landslide susceptibility, land use, and area type (urban or rural).

In order to identify the most relevant input features as well as reduce the computational cost and the complexity of MLAs, three wrapper feature selection approaches (forward, backward, and bi-direction wrapper) have been exploited [94,95,96]. After the process, it has been demonstrated that the backward wrapper showed the highest performance and allowed us to identify the optimal set of input features, composed by nine factors: elevation, average yearly rainfall, distance from rivers, distance from landslides, earthquake susceptibility, area type, river density, silt content in the subsoil, and clay content in the subsoil.

In order to train and test the MLAs, a random split of the dataset in 70% of samples for the training phase and 30% of samples for the test phase has been performed. The highest level of generalization has been achieved by implementing a 10-Fold Cross-Validation [97] during the training phase. The hyper-parameter tuning phase of each algorithm has been performed according to a Bayesian Optimization Algorithm [98].

**Figure 7:**
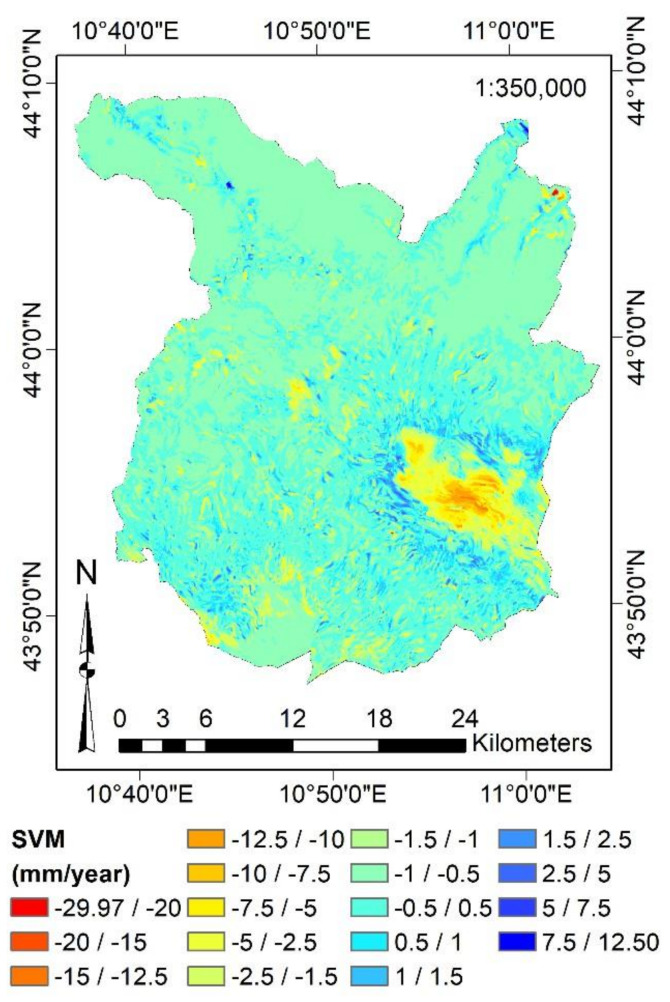
Surface motion mapping over the study area by SVM. The present figure has been adapted from Fiorentini et al. [8].

Finally, the MLAs have been evaluated by several numerical and graphical metrics, such as Correlation Coefficient (R^2^), Root Mean Square Error (RMSE), Mean Absolute Error (MAE), Scatter Plot, and Taylor Diagram [99]. Outcomes showed that SVM and BRT are the most suitable algorithms for predicting surface motion. BRT showed the highest R^2^ (0.96) and the lowest RMSE (0.44 mm/year), whereas SVM reported the lowest difference between the standard deviation of its predictions (2.05 mm/year) and that of the reference samples (2.09 mm/year). By observing the Taylor Diagram, we presumed that the SVM provides the highest performance. Figure 7 shows the predicted subsidence effect for the entire study area, computed by the SVM.

Figure 7 demonstrates that the SVM algorithm can reliably predict the subsidence of the urban area of Pistoia (yellow and orange areas) as well as the surrounding swelling effects (blue areas). Furthermore, it allows mapping the surface movements in every point of the Province of Pistoia, even where there were no PS. The resolution of the cells reporting the predictions of the SVM algorithm is 10 × 10 m.

Subsequently, thanks to the superimposition of the road graph, and under the hypothesis that displacements detected by the InSAR analysis could translate into surface deformations of infrastructures, all the surface movements in each point of the infrastructure were mapped. The reliability of such a hypothesis has been demonstrated by statistical metrics and four on-site inspections [7,8].

**Figure 8:**
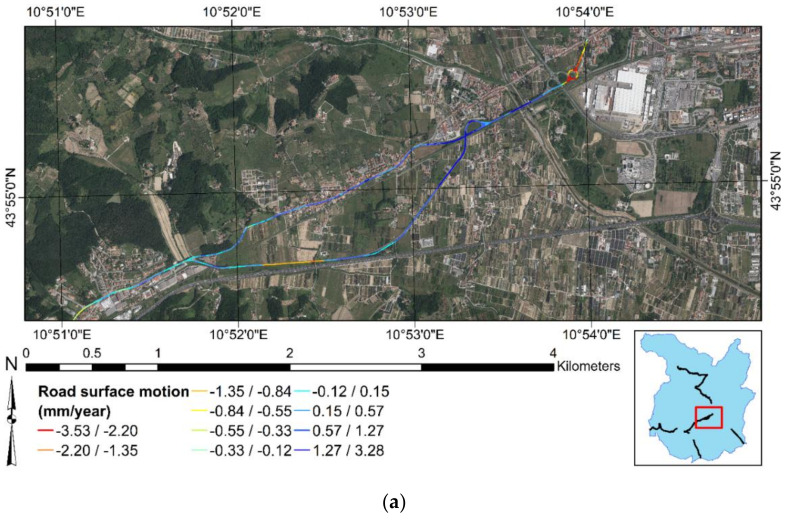

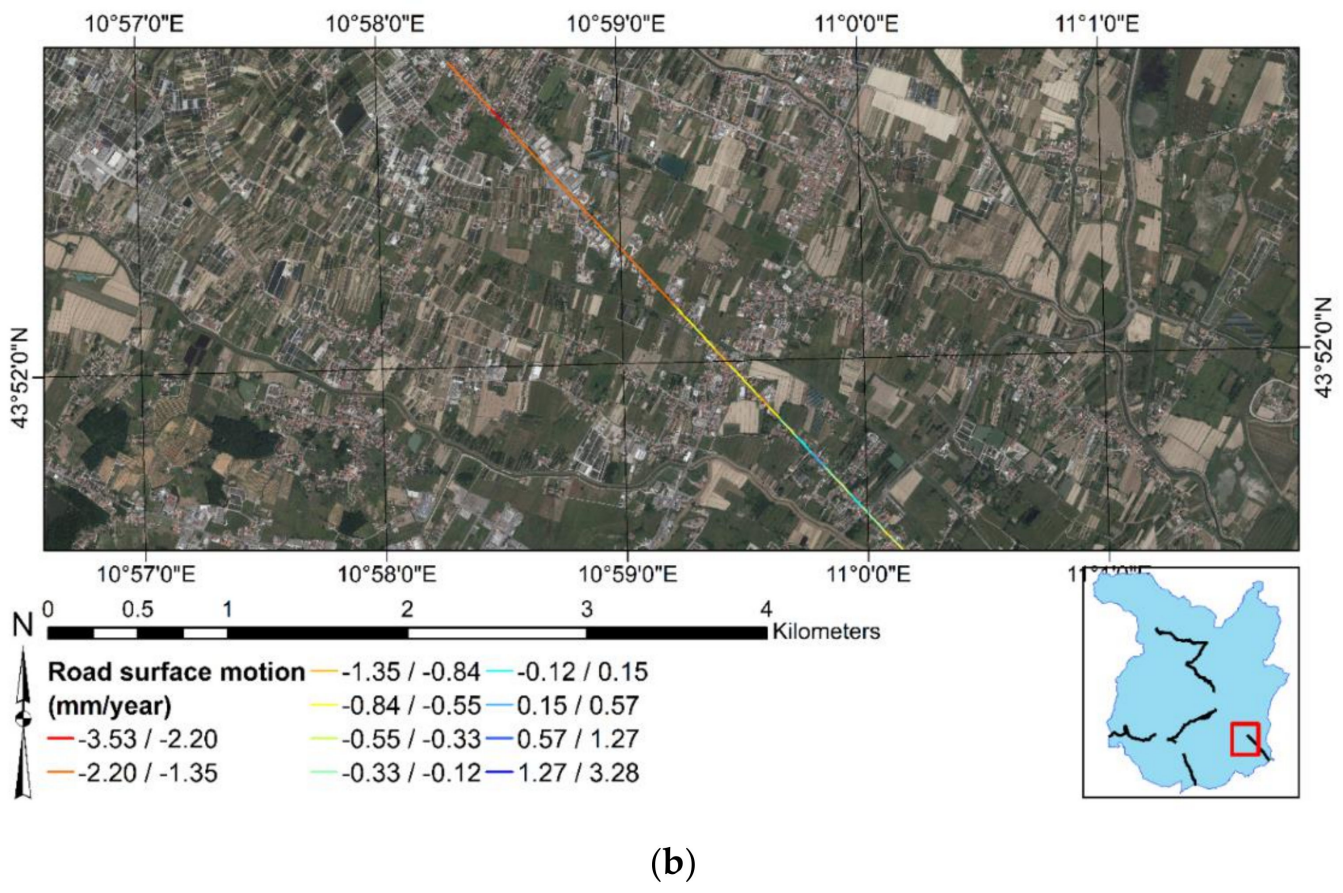
Road surface deformation estimated by MLAs: (**a**) test site 1 and test site 2. (**b**) Test site 3. The present figure has been adapted from Fiorentini et al. [8].

Figure 8 recaps the outcomes of MLAs focused on the test sites of the present research. Figure 8a is related to test site 1 and test site 2, while Figure 8b highlights the road surface condition of test site 3. It is shown that most of the negative displacements are occurring at the end of test site 2. There is a roundabout intersection that reports deformations higher than −2.2 mm/year. In addition, Figure 8a indicates that most of the positive displacements occur at the end of test site 1, with an intensity of more than +1.3 mm/year. As for test site 3, Figure 8b indicates that MLAs predict negative displacements for the whole stretch, with increasing intensity of deformation from the South to the North direction.

It is worth mentioning that road deformations are derived from a raster-based output map whose cells have a resolution of 10 × 10 m.

## 4. Methodology

### 4.1. Workflow

**Figure 9:**
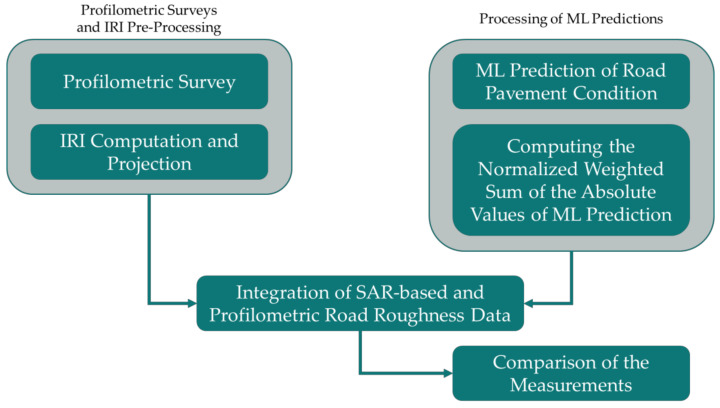
Workflow of the proposed methodology.

The main steps of the proposed procedure have been recapped in the flowchart shown in Figure 9.

First, there are two main phases: (1) profilometric surveys and IRI pre-processing, and (2) processing of ML predictions.

- The former phase indicates the necessity to carry out the profilometric surveys by a laser profilometer. In the present surveys, a laser profilometer has been employed for surveying three test sites, for an overall length of 10 km. Once the surveys are done, the IRI has been computed and projected geographically along with the test sites;
- The second main phase involves using a long stack of SAR images to exploit the PS-InSAR technique and identify where and how many roads are affected by displacements connected to exogenous factors. The PS-InSAR technique has a point-based outcome. If a medium-resolution satellite (such as Sentinel-1) is exploited, there are large areas of roads with no PS. Therefore, the calibration of MLAs able to correlate several environmental parameters and displacement detected by the SAR sensor is essential for having a complete coverage of deformation estimates over the tested roads. Such MLAs have been calibrated in References [7,8]. In the present paper, their outcomes need to be processed. To compare MLAs outcomes with the IRI values, the computation of the normalized weighted sum of the absolute values of ML predictions is necessary.

Once these two main phases have been concluded, we proceeded with the comparison between the two measurements, verifying whether there is a correspondence.

### 4.2. IRI Computation by Profilometric Measurements

**Figure 10:**
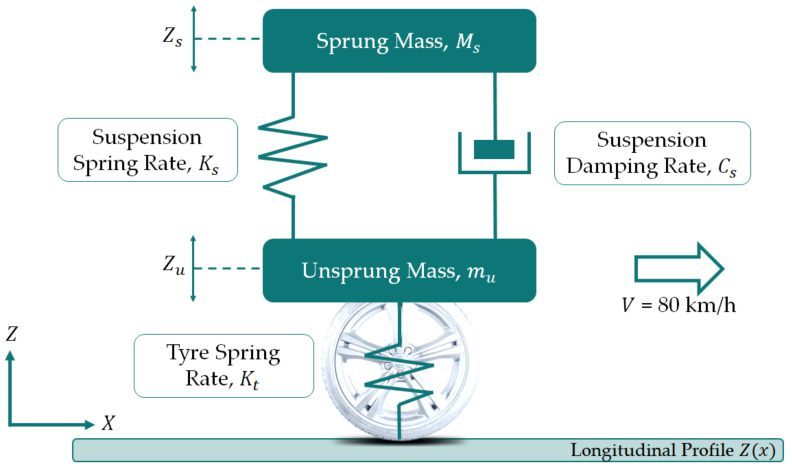
The quarter car model.

The IRI is a dimensionless parameter, usually expressed in [m/km] or [mm/m], which assumes a value equal to zero for a perfectly linear profile and grows as the irregularities increase. The calculation procedure consists of simulating, using a mathematical model called Quarter Car Model (QCM), the passage of a quarter of a vehicle at a speed of 80 km/h on the measured profile, and evaluating the cumulated displacements of the suspensions (Figure 10). The body of the vehicle behaves like a suspended mass positioned above a suspension. The latter is modeled by employing two elements: one elastic and one damping. The suspension is then connected to an un-sprung mass (wheel, brake system, and suspension components). The contact between the wheel and the irregularities of the road surface inflects the tire and sets the suspension in motion, causing vibrations in the vertical direction of both the un-sprung and suspended masses.

Accordingly, the IRI index corresponds to the accumulation of relative displacements between the suspended mass and the un-sprung mass of the QCM, averaged by the length of the analyzed profile. Mathematically, the IRI index can be expressed using the following equation (Equation (1)).

(1) $$IRI=\frac{1}{l}\cdot \overset{l/S}{\underset{0}{\int }}\left|\dot{Z_{s}}-\dot{Z_{u}}\right|dt$$

where IRI could be expressed in [m/km], l is the length of the profile in km, S is the simulated speed of the QCM (80 km/h),$\;\dot{Z_{s}}$ is the time derivative of the height of the sprung mass, and $\dot{Z_{u}}$ is the time derivative of the height of the unsprung mass.

Concerning the AASHTO Designation PP37 [100], the IRI is determined as follows:

- The IRI is calculated from a single longitudinal profile. The general recommendation is (a) to carry out the measurements of the profile along the alignments corresponding to the tire tracks, (b) proceed with the calculation of the IRI every 100 m of the profile, and (c) carry out an average of two values in order to have a representative value of the alignment irregularities;
- The measured profile is filtered by the moving average method on a baseline of 250 mm (this filter should be omitted if the profile has already been filtered by a moving average or an anti-aliasing filter that attenuates wavelengths smaller than 500 mm);
- The filtered profile is subjected to a further filter represented by the QCM with the following parameters (Table 3).

The QCM parameters have been defined in Figure 10.

- The simulated movement of the suspension, i.e., the amplitude of the displacement between the suspended mass and the un-sprung mass that occurs in the QCM, is linearly accumulated (by adding the absolute values of the displacements);
- The accumulation of displacements is normalized on the length of the profile.

The primary sensor of the laser profiler is a digital Selcom laser profile, which measures the distance to the road in order to produce a longitudinal profile. This data is synchronized with input from a highly precise odometer sensor and an accelerometer. By processing the data recorded during the survey and determining the IRI, information can be obtained regarding the Mean Profile Depth [101,102] and the Estimated Texture Depth [103,104], which are indicators of the macro-texture of the road pavement surface. Table 4 summarizes the laser profiler characteristics and sampling concerning the parameters defined by the standard ISO 13473-3 [105].

The survey of the longitudinal profiles was performed following the procedure reported in the Standard ISO 13473-1 [106]. The acquisition speed was set to 40 km/h. The World Bank [47] procedure for computing the IRI has been implemented in MATLAB 2020b [107]. Furthermore, by using ESRI ArcGIS 10.5, the outcomes have been projected along the test sites to be compared with the estimations provided by MLAs.

### 4.3. Normalized Weighted Sum of the Absolute Value of ML Predictions

It has been said that IRI measurements are expressed in [mm/m], while MLAs estimates are expressed in [mm/year]. Additionally, IRI measurements are sampled every 50 mm (according to the laser profiler characteristics) and integrated over 100 m, while ML predictions are derived from a raster-based map with a resolution of 10 × 10 m. Finally, IRI can be only positive (from zero onwards), while ML predictions can be both positive and negative. Consequently, the comparison between these two parameters is not immediate and requires the computation of the normalized weighted sum of the absolute values of the ML predictions (Equation (2)).

(2) $$ML_{NWSA}=\frac{1}{L}\cdot \overset{n}{\underset{i=1}{\sum }}\left|ML_{i}\right|\cdot l_{i}$$

where $ML_{NWSA}$ is the normalized weighted sum of the absolute values of the ML predictions. The parameters $\left|ML_{i}\right|,\;l_{i}$, n, and L can be computed according to the following steps.

- **Computation of the absolute values**: Considering that the ML predictions can be both positive and negative, consistently with the calculation of IRI (Equation (1)), the absolute value, $\left|ML_{i}\right|$, is computed (Equation (2)) for each ML prediction, under the assumption that positive (swelling) and negative (subsidence) deformations have the same importance for assessing the quality of road regularity;
- **Road association and computation of the weighted value**: the absolute value of ML prediction related to each cell is associated with the correspondent road stretch that crosses it. Subsequently, this value is multiplied by the length of the road stretch considered, $l_{i}$ (Equation (2));
- **Computation of the weighted sum**: according to the computation of the IRI, the weighted value of ML predictions is cumulated every 100 m for each 100-m section on which the IRI parameter has been calculated;
- **Normalization:** consistently with the IRI, the weighted sums are divided by the length of the road section, L, i.e., by 100 m (Equation (2)). In this way, the weighted sum of the absolute value of ML predictions are sampled for road sections of 100 m in length.

**Figure 11:**
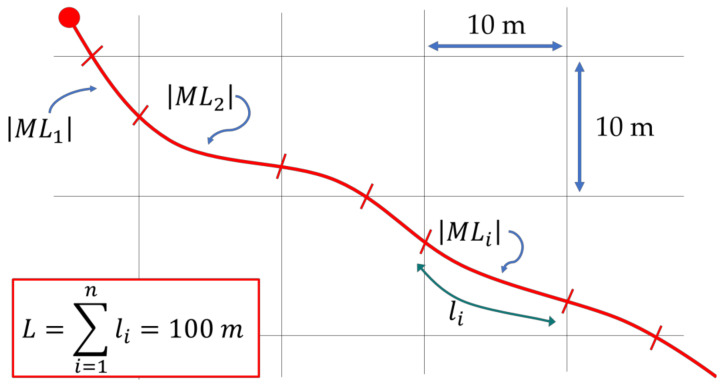
Computation of $ML_{NWSA}$.

Figure 11 graphically explains the definition of the terms in Equation (2). The black lines represent the raster grid of the output map derived from MLAs and calibrated in References [7,8]. In each cell, an absolute value of ML prediction insists. The red line represents the axis of a road section that is crossing the area.

Accordingly, even if the two parameters (IRI and $ML_{NWSA}$) have a different scale of values, they are proportionate. They can assume only positive values, and lower values of both indicate road pavement in good conditions, whereas high values of both indicate poor quality of road pavements. Moreover, both ML predictions and IRI values are computed for the same road sections of 100 m in length. Accordingly, the two measurements should be more easily comparable.

### 4.4. In Situ Comparison of the Outcomes

Once the IRI parameter and $ML_{NWSA}$ have been calculated, it is possible to perform a comparison of the two values for each surveyed road section. For this purpose, a specific plot has been defined for each test site. On the abscissa axis, it shows the longitudinal progressives of the road stretch, while, on the ordinate axis, it shows the two parameters to be compared (IRI and $ML_{NWSA}$). This graph should verify the areas with greater correlation and lesser correlation, distinguishing their peculiarities.

## 5. Results and Discussion

**Figure 12:**
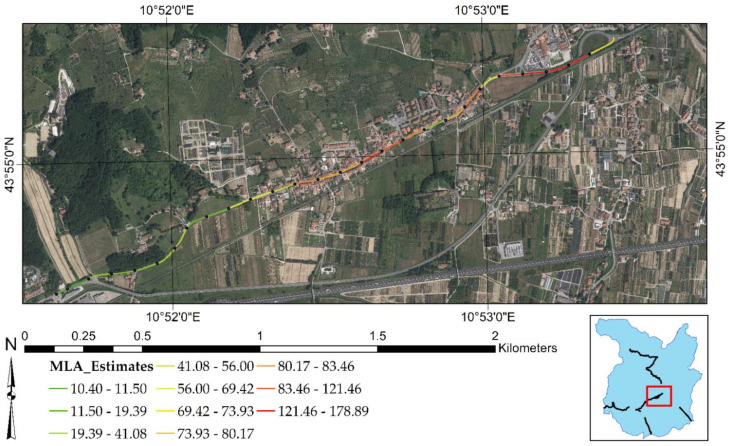
Normalized weighted sum of the absolute value of ML predictions: test site 1.

**Figure 13:**
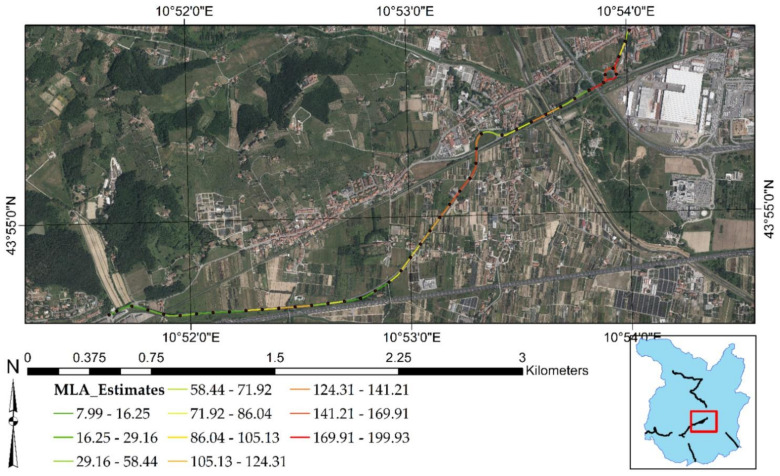
Normalized weighted sum of the absolute value of ML predictions: test site 2.

**Figure 14:**
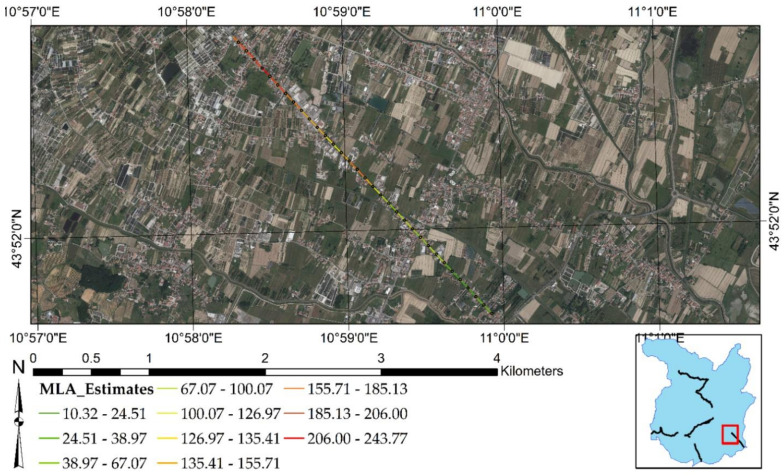
Normalized weighted sum of the absolute value of ML predictions: test site 3.

Figure 12 (test site 1), Figure 13 (test site 2), and Figure 14 (test site 3) show the $ML_{NWSA}$ for stretches of 100 m in length. We can appreciate their estimation in a positive scale of values from zero onwards, where zero corresponds to perfect conditions of the pavement, and increasing values correspond to progressively worse pavement conditions.

Figure 12, Figure 13 and Figure 14 clearly reveal that the $ML_{NWSA}$ computation did not impact the surface motion predicted by MLAs, preserving the degree of criticality. The demonstration of this aspect derives from a direct comparison with the surface motion predicted by MLAs highlighted in Figure 3.

Regarding test site 1 (Figure 12), the highest positive surface motions (uplifts) are identified at the end of the road stretch. Concerning test site 2 (Figure 13), the major negative deformations (subsidence phenomena) are detected at the end of the road stretch (at the roundabout intersection). Finally, in test site 3 (Figure 14), the deformations increase as one approaches the city of Pistoia, i.e., reaching the final part of the road stretch toward the Northwest. The same criticalities have been highlighted in Figure 3. Consequently, neither the new scale of values of $ML_{NWSA}$ nor the new 100-m-based resampling should influence the ML predictions.

**Figure 15:**
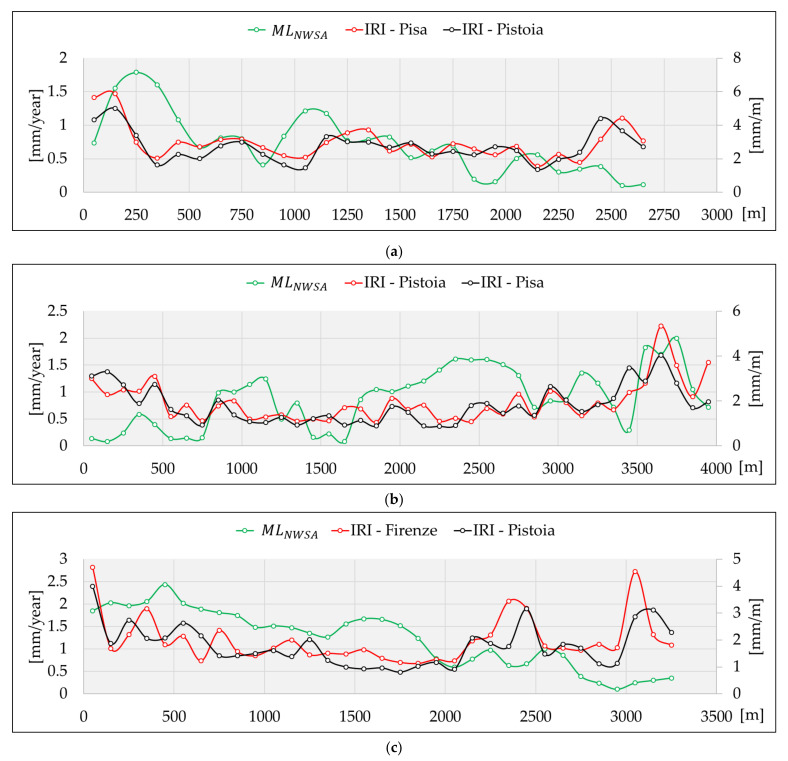
Comparison between IRI and the normalized weighted sum of the absolute value of ML predictions: (**a**) test site 1, (**b**) test site 2, and (**c**) test site 3.

Accordingly, once the $ML_{NWSA}$ have been computed, a comparison with the IRI values is carried out by specific plots. Figure 15 focuses on this aspect by showing a comparison plot for each test site. The reference travel direction (according to labels reported in Figure 3) is Pisa for test site 1 (Figure 15a), Pistoia for test site 2 (Figure 15b), and Florence for test site 3 (Figure 15c). The abscissa axis reports the longitudinal progressives of the road stretches. The profilometric surveys have been reported by a solid red line and a solid black line. We employed two different colors since laser profilometric surveys have been carried out for each lane of the test sites. An IRI value has been computed for each lane of the carriageway. Moreover, it is worth mentioning that each IRI value represents the average of two measurements. For each lane of the carriageway, two profilometric surveys have been carried out. Finally, a solid green line represents values of the $ML_{NWSA}$.

**Figure 16:**
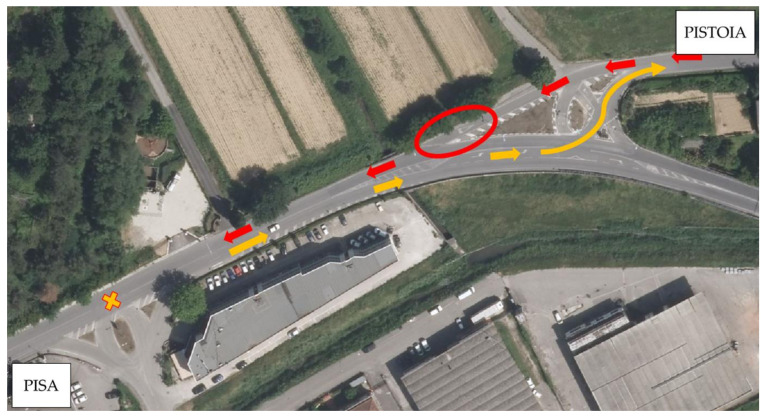
Focus on the paths followed by the laser profiler in test site 1.

Figure 15 demonstrates that the trends are comparable and that there is a certain correlation between profilometric measurements and ML predictions. Specifically, Figure 15a is the one that best represents this correlation. There is an initial peak in the roughness values correctly identified by the MLAs. Moreover, there is a second ascent zone around 1100 m, which is also identified by increasing ML predictions. After that, the IRI progressively decreases to approximately 2300 m, and so do the ML predictions. At the end of test site 1, we can appreciate an inconsistency between the two measures. Investigating the area (Figure 16), we can see the presence of a road intersection that forces the vehicle to take an S-shaped path in one direction of travel (Pistoia) and to a stop with a right-of-way in the other (Pisa). These two factors may have affected the IRI value of both directions since the driver is not able to maintain the velocity of 40 km/h (in both directions) steadily, and horizontal acceleration has a significant effect (in Pistoia direction). These facts may result in a distortion of the real condition of road regularity. With regard to Figure 15b, a u-shaped trend of the profilometric measurements is observed. The ML predictions show a fluctuating trend with low deformation values in the decreasing part (between 0 and 1500 m). After 1500 m, the IRI values grow, and the ML predictions grow consistently. The road sections in the worst condition are at the end of test site 2 (3600 to 3800 m). In this part, the ML predictions are still consistent and appropriately identify the worst conditions of the stretch. Finally, with regard to Figure 15c, only a qualitative association can be detected. IRI observations fluctuate between sections (for example, between 0 and 800 m, and from 2100 m to the end of the segment). There is a general decrease in IRI values from the beginning of the stretch up to 2100 m. In this first part of the segment, the trend of the ML predictions agrees. Subsequently, the IRI measures show a general increase, while the ML predictions continue to decrease. In this area, there is no agreement between the two measurements.

These considerations lead us to support the following relevant aspects.

- When the profiles (IRI and $ML_{NWSA}$) are similar, the dependence between the two measurements is conceivable. In these cases, we could expect that the deformations of the road pavement are a result of exogenous events, which MLAs considered during their training. Accordingly, when road regularity is driven by natural occurrences, these algorithms are able to replace in situ surveys, allowing road authorities to estimate the road pavement condition with a sufficient degree of reliability;
- When the two profiles are not similar, we could suppose that the IRI pattern is a consequence of factors that the MLAs do not consider, such as endogenous factors. Such factors are related to the inherent features of the infrastructure (layer structure, traffic flow, age of the pavements, etc.) and may affect road regularity. It would be necessary to carry out further specific analyses of road pavements with other types of NDT-based surveys (e.g., Falling Weight Deflectometer and Ground Penetrating Radar) to recognize the actual causes that affect pavement regularity.

As said previously, it should also be considered that the IRI measurement may be affected by some sources of error. IRI is related to the quality of the profilometric survey, and the following aspects deserve to be underlined.

- The survey should be continuously performed at 40 km/h. The traffic flow, the presence of pedestrian crossings, road intersections, driveways, and any obstacles along the way could force the operator to alter his speed. This issue is relevant, especially in the urban context. To reduce it, we chose to carry out the surveys at night, on a non-holiday day. Nonetheless, it cannot be excluded that these aspects may have influenced the surveys;
- There are some sections in rather tight curves (e.g., the section of test site 1 highlighted in Figure 16 and the roundabout at the end of test site 2), which constitute areas of potential error of the IRI measurement since the horizontal accelerations are not measured and compensated by an accelerometer. Such an acceleration could increase the IRI value. Moreover, in these sections, keeping the speed of the profilometer steady is complicated;
- Large areas subjected to subsidence could not be detected by the profilometer because they assume wavelengths longer than those of the laser sensor.

Finally, it should be considered that the association between IRI and $ML_{NWSA}$ could differ since the ML prediction derives from an interferometric process of a medium-resolution satellite operating in a C-band. The calibration of MLAs by X-band SAR images could allow mapping the surface motion of infrastructures with greater reliability, being able to exploit a denser and higher resolution data [4,6]. In any case, having calibrated the MLAs by building pixels of 10 × 10 m around a considerable number of PS (52,257), this is not guaranteed or proven. Nonetheless, we have considered this eventuality, and we are currently requesting high-resolution SAR images to see if they can improve the results obtained in this research.

Road authorities and practitioners may implement the present procedure in a GIS environment to easily and expeditiously obtain useful data on road regularity concerning vast and heterogenous infrastructural networks, even though such information could only be a first glance at the quality of the managed network. When there are scarce funds available, and there are numerous road sections to investigate, employing this strategy could have sound impacts on the dynamics of the decision-making processes of the road authorities. On the one hand, it is possible to restrict the range of inspections to targeted sites that have highlighted the highest criticalities. On the other hand, it is possible to carry out planning activities and evaluate which areas are most suitable for hosting an infrastructure whose quality should last over time. Finally, it is possible to operate with prevention activities. It is possible to set specific environmental features as the input of MLAs to verify the consequences on the regularity of the road pavements. Therefore, starting from environmental data and PS-InSAR outcomes derived from medium-resolution SAR measurements of large areas, Machine Learning Algorithms can be calibrated efficiently to evaluate the vertical displacements in every point of a road pavement. Accordingly, it is possible to evaluate the regularity of road pavements by using a proxy value of the IRI parameter with a certain degree of approximation.

## 6. Conclusions

Can Machine Learning and PS-InSAR reliably stand in for road profilometric surveys? The question arises at the beginning of this paper and highlights its main purpose: in the field of infrastructural surveys, is it possible to correlate these two survey techniques? This would certainly have advantages. First of all, road authorities could have a tool capable of carrying out assessments on the state of a road network, including even extensive ones, in an automatic, expeditious, and efficient manner. In addition, it would result in a substantial saving of time and money.

We have tried to face this ambitious challenge through the joint use of three essential components: (1) SAR image processing by means of the PS-InSAR algorithm, (2) laser profilometer surveys and IRI computation, and (3) Machine Learning modeling. The PS-InSAR algorithm exploited 210 co-registered medium-resolution SAR images of the SAR sensor mounted on Sentinel-1. We chose Sentinel-1 as the SAR data can be immediately supplied to road authorities free of costs. Furthermore, in the case of the Tuscany Region, these data are processed every new Sentinel-1 acquisition, and made available on a specific online platform, which allows viewing, analysis, and downloading of the data. The use of medium-resolution SAR data involves limitations that must be alleviated. Since the present procedure is applied to rural and urban roads with two lanes and a single carriageway, the number of PS falling on infrastructures is not high and, consequently, infrastructures are not fully mapped. On the other hand, the laser profilometric survey (and related IRI calculation) allows us to evaluate the road surface regularity in each point of the infrastructure, but it is an expensive survey, both in terms of time and money. Furthermore, it involves interference with circulating traffic, increasing the possibility of measurement errors. Therefore, the proposed methodology is based on the use of Machine Learning modeling to map the surface motion (detected by the PS-InSAR survey) in each point of an infrastructure. To this end, in two of our previous works, five Machine Learning Algorithms have been proposed that allow effective correlations of the PS-InSAR measurements to the topographical, hydrological, geological, and geomorphological characteristics of a site.

In order to correlate these two infrastructural surveys, 10 km of a laser profilometer survey were carried out on three different test sites, including two in a rural area and one in an urban area. Subsequently, the two measurements (outcomes of PS-InSAR process and outcomes of a laser profilometer survey) have been homogenized.

Significant correlations were observed between the conventional IRI road roughness measurements and the MLA predictions involving PS-InSAR data. We interpreted these correlations as being influenced primarily by exogenous (external) factors such as large-area subsidence. Conversely, the areas of weak correlation were deemed to be governed by endogenous (local) factors associated with the properties of road materials, pavement section structure, and traffic loading. Calibrating MLAs with input features representative of endogenous conditions will be the focus of future research aimed at integrating satellite remote sensing of the transportation infrastructure and other NDT-based surveys by Machine Learning.

## Figures and Tables

**Table 1:** Machine and deep learning algorithms in road pavement management.

Reference	Algorithms	Output	Road	Pavement	Data
Hossain et al. [56]	ANN	IRI	FHWA LTPP program	Flexible pavements	Climate and traffic data
Choi et al. [57]	RNN	IRI after 1 year, Percentage of Cracks, Rutting Depth	RPM data from the Korean NHPMS	/	Climate, traffic, and de-icing data
Kargah-Ostadi et al. [58]	ANN	IRI	FHWA LTPP program	Flexible rehabilitated pavements	Climatic and traffic data, previous and pre-overlay IRI, road intervention data, subgrade data
Kargah-Ostadi et al. [59]	ANN, RBFNN, SVM	IRI	FHWA LTPP program	Flexible rehabilitated pavements	Use of principal component analysis of data gathered in Reference [58]
Gong et al. [60]	RF, RLR	IRI	FHWA LTPP program	Flexible pavements	Distress measurements, traffic, climatic, maintenance, and structural data
Abd El-Hakim et al. [61]	ANN	IRI	FHWA LTPP program	Jointed plain concrete pavements	Initial IRI value, pavement age, pavement distress data, and percent subgrade passing No. 200 U.S. sieve
Ziari et al. [62]	ANN, GMDH	IRI after 1 year, IRI after 2 years, and IRI at the end of pavement life cycle	FHWA LTPP program	Flexible pavements without rehabilitations	Structural, Climatic, and traffic data
Marcelino et al. [63]	RF	IRI after 5 years, IRI after 10 years	FHWA LTPP program	/	Previous IRI values, structural, climatic, and traffic data
Marcelino et al. [64]	CART-BRT, RF-BRT	IRI	FHWA LTPP (training) PRA (testing)	Flexible pavements	IRI, traffic data, pavement thickness, climate data, structural number
Kaloop et al. [65]	WOPELM, regression curves, ANN	IRI	FHWA LTPP program	Jointed plain concrete pavements	Initial IRI value, pavement age, pavement distress data, and percent subgrade passing No. 200 U.S. sieve
Abdelaziz et al. [66]	MLR, ANN	IRI	FHWA LTPP program	Both original and overlaid flexible pavements	Initial IRI value, pavement age, transverse cracks, alligator cracks, and the standard deviation of the rut depth
Lin et al. [67]	ANN	IRI	THB	/	Pavement distresses identified in video images recorded by a camera mounted on ARAN
Kargah-Ostadi et al. [68]	ANN, RBF, SVM	IRI	FHWA LTPP program	Flexible pavements with varying structural factors and alternative rehabilitation treatments	Climatic and traffic data, previous IRI, road intervention data, subgrade data
Mazari et al. [69]	GEP-ANN	IRI	FHWA LTPP program	Flexible pavement over unbound granular layers	(1) Traffic and structural data, and (2) initial IRI value initial age, initial cumulative ESAL, the difference in age, and the difference in cumulative ESAL

Acronyms: ANN = Artificial Neural Network, RNN = Recurrent Neural Network, RBFNN = Radial Basis Function Neural Network, SVM = Support Vector Machine, RLR = Regularized Linear Regression, GMDH = Group Method of Data Handling, CART-BRT = Classification and Regression Tree-Based Boosted Regression Tree, RF-BRT = Random Forest-Based Boosted Regression Tree, WOPELM = Weighted-Optimally-Pruned Extreme Learning Machine, MLR = Multiple Linear Regression, GEP-ANN = Gene Expression Programming—ANN, FHWA LTPP = Federal Highway Administration Long-Term Pavement Performance Database (https://highways.dot.gov/research/long-term-infrastructure-performance/ltpp/long-term-pavement-performance, accessed on 12 February 2021), RPM data from Korean NHPMS = Road Pavement Monitoring data from the Korean National Highway Pavement Management System, PRA = Portuguese Road Administration, THB = Taiwan Highway Bureau, ARAN = Automatic Road Analyzer, ESAL = Equivalent Single Axle Load.

**Table 2:** Geometry, functional, structural, and local geotechnical information of test sites.

	Unit	Rural, “C1”	Urban, “E1”
Lane width	[m]	3.25 ÷ 3.75	2.75 ÷ 3.25
Pavement Shoulder width	[m]	1.0 ÷ 1.50	0.3 ÷ 0.5
Sidewalk width	[m]	/	1 ÷ 1.50
AADT	[vehicles/day]	7000	12,500
Percentage of trucks	[%]	10.0 ÷ 12.0	3.0 ÷ 5.0
Asphalt concrete layers, $H_{1}$	[cm]	15 ÷ 20	15 ÷ 20
Subbase course, $H_{2}$	[cm]	40 ÷ 50	25 ÷ 30
$E_{m}$ at 20 °C for asphalt concrete	[MPa]	4500 ÷ 5500	4000 ÷ 5000
E for sub-base	[MPa]	200 ÷ 300	400 ÷ 500
E for subgrade	[MPa]	80 ÷ 100	70 ÷ 90
Subgrade—AASHTO Soil Classification System	[-]	A5, A6, A7-5 silty soils and clayey soils	A5, A6, A7-5 silty soils and clayey soils

**Table 3:** QCM parameters.

V	$\frac{K_{s}}{M_{s}}\;$	$\frac{K_{t}}{M_{s}}\;$	$\frac{C_{s}}{M_{s}}\;$	$\frac{m_{u}}{M_{s}}\;$
[km/h]	[1/s^2^]	[1/s^2^]	[1/s]	[-]
80	63.3	653	6	0.15

**Table 4:** Characteristics of the laser profiler and sampling.

Device	Wavelength	Sampling Interval	Vertical Resolution	Laser Beam Frequency	Acquisition Speed
Contactless	D–F	50 mm	0.05 mm	16 kHz	40–60–80 km/h

## Data Availability

Not applicable.
